# Ndrg1 promotes adipocyte differentiation and sustains their function

**DOI:** 10.1038/s41598-017-07497-x

**Published:** 2017-08-03

**Authors:** Kai Cai, Rabih El-Merahbi, Mona Loeffler, Alexander E. Mayer, Grzegorz Sumara

**Affiliations:** Rudolf Virchow Center for Experimental Biomedicine, University of Würzburg, Josef-Schneider-Str. 2, Haus D15, D-97080 Würzburg, Germany

## Abstract

Adipocytes play a central role in maintaining metabolic homeostasis in the body. Differentiation of adipocyte precursor cells requires the transcriptional activity of peroxisome proliferator-activated receptor-γ (Pparγ) and CCAAT/enhancer binding proteins (C/Ebps). Transcriptional activity is regulated by signaling modules activated by a plethora of hormones and nutrients. Mechanistic target of rapamacin complexes (mTORC) 1 and 2 are central for the coordination of hormonal and nutritional inputs in cells and are essential for adipogenesis. Serum glucocorticoid kinase 1 (Sgk1)-dependent phosphorylation of N-Myc downstream-regulated gene 1 (Ndrg1) is a hallmark of mTORC2 activation in cells. Moreover, Pparγ activation promotes Ndrg1 expression. However, the impact of Ndrg1 on adipocyte differentiation and function has not yet been defined. Here, we show that Ndrg1 expression and its Sgk1-dependent phosphorylation are induced during adipogenesis. Consistently, we demonstrate that Ndrg1 promotes adipocyte differentiation and function by inducing Pparγ expression. Additionally, our results indicate that Ndrg1 is required for C/Ebpα phosphorylation. Moreover, we found that Ndrg1 phosphorylation by Sgk1 promotes adipocyte formation. Taken together, we show that induction of Ndrg1 expression by Pparγ and its phosphorylation by Sgk1 kinase are required for the acquisition of adipocyte characteristics by precursor cells.

## Introduction

White adipocytes are central for the regulation of lipid and metabolic homeostasis^1^. Importantly, both deficits in adipose tissue development (lipodystrophy) and the excessive accumulation of adipose tissue result in metabolic disorders, including type 2 diabetes^2^. Differentiation of mesenchymal precursor cells into adipocytes is crucial for adipose tissue acquisition^2^. The terminal differentiation of adipocytes requires the coordinated expression of genes regulating their specific functions. Peroxisome proliferator-activated receptor-γ (Pparγ) and CCAAT/enhancer binding proteins (C/Ebps) are major transcription factors promoting the acquisition of molecular adipocyte characteristics by precursor cells^2^. A plethora of extracellular signals, including hormones and nutrients, activate intracellular signaling cascades that modulate Pparγ, C/Ebps and other transcription factors to regulate adipogenesis^3^.

Mechanistic target of rapamycin (mTOR) activity is dependent on both hormones (insulin) and nutrients levels^4^. In cells, mTOR kinase is present in two large multi-component signaling complexes; mTORC1 and mTORC2 (mTOR complex 1 and 2). mTORC1 is defined primarily by raptor protein, while mTORC2 by the presence of the rictor subunit. Depletion of raptor specifically abrogates the activity of mTORC1, while rictor is specifically required for mTORC2 function^4^. Genetic and pharmacological studies revealed that both mTORC1 and mTORC2 are required for adipogenesis and promote adipocyte function^5–8^. Yet, the function of distal mTORC effectors in the regulation of adipocyte formation is not completely understood.

mTORC2 activates Sgk1, which is required for adipocyte formation^9–12^. Ndrg1 is a major phosphorylation target of Sgk1^13–15^. Moreover, expression of Ndrg1 in adipocytes seems to be promoted by Pparγ^16, 17^. Ndrg1 was previously implicated in regulation of progression of multiple tumors^18–22^, peripheral neuropathy^23, 24^, T cell anergy^25^, as well as bone remodeling and macrophage differentiation^26^. Additionally, a number of studies suggest that Ndrg1 might regulate other signaling cascades that influence adipocyte differentiation. This includes the Wnt/β-Catenin cascade, glycogen synthase kinase 3β (Gsk3β), as well as extracellular regulated kinase 1/2 (Erk1/2)^3, 27–29^. However, the impact of Ndrg1 on the regulation of adipogenesis and adipocyte function has not been elucidated. Here, we show that Ndrg1 promotes adipogenesis and sustains adipocyte function by promoting Pparγ expression and possibly C/Ebpα activity. Moreover, our results suggest that Sgk1-dependent phosphorylation of threonine 346 on Ndrg1 promotes adipocyte differentiation.

## Results

### Ndrg1 is induced during adipocyte differentiation

**Figure 1:**
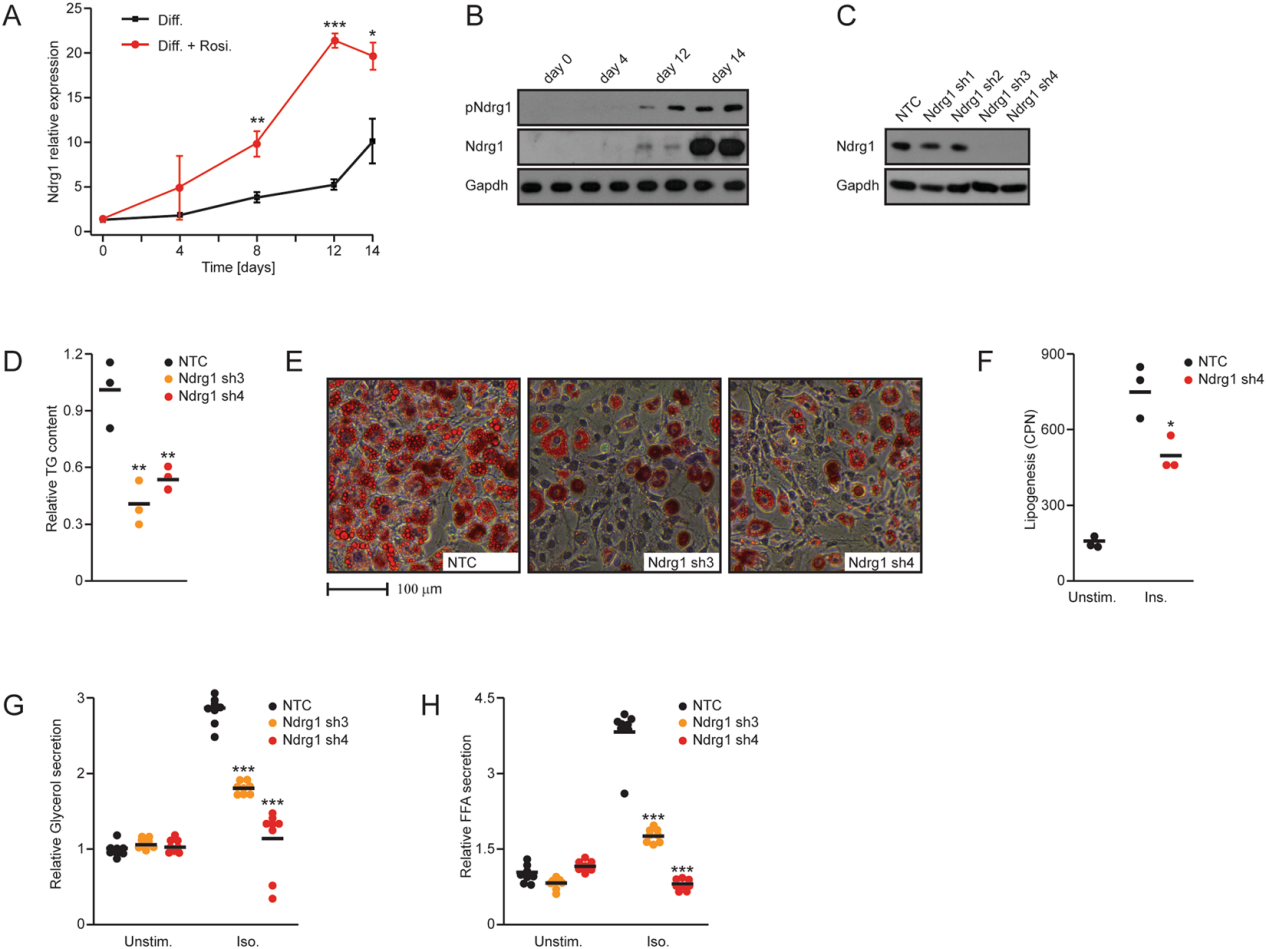
Ndrg1 promotes adipocyte differentiation and lipolysis. (**A**) Expression of Ndrg1 mRNA in 3t3l1 cells at different stages of differentiation with or without rosiglitazone (Rosi). Stars indicate significance between expression levels in control and Rosi-treated cells at the specific time points (*P < 0.05, **P < 0.01, ***P < 0.001 according to a t-test). (**B**) Total and phospho-Ndrg1 levels at different stages of 3t3l1 differentiation. (**C**) Ndrg1 protein levels in cells targeted by different shRNAs against Ndrg1 (Ndrg1 sh). (**D**) Relative TG content and (**E**) neutral lipid staining (OilRedO) on differentiated 3t3l1 cells after knockdown of Ndrg1. (**F**) Lipogenesis rate in control and Ndrg1 sh cells, unstimulated (unstim.) or upon insulin (Ins.) stimulation. (**G**) Relative glycerol and (H) FFA levels in medium from differentiated 3t3l1 control cells or Ndrg1 knockdown cells stimulated with control medium or isoproterenol (Iso). For all graphs, each data point represents a biological replicate. Stars indicate significance for given parameters between control and Ndrg1-depleted cells. (*P < 0.05, **P < 0.01, ***P < 0.001 according to ANOVA followed by the Post hoc Tukey test).

To test whether Ndrg1 could regulate adipocyte differentiation, we first measured its expression in the 3t3l1 pre-adipocyte cell line, which was subjected to an adipocyte-differentiation cocktail for different time points. Expression of Ndrg1 mRNA increased progressively during differentiation, reaching more than 10-fold elevation in fully differentiated cells compared to undifferentiated 3t3l1 cells (Fig. 1A). Previous studies indicated that Ndrg1 is a target of the master transcription factor promoting adipogenesis – Pparγ^16, 17^. In fact, 3t3l1 cells differentiated in the presence of the Pparγ agonist rosiglitazone present markedly higher expression of Ndrg1 after 8, 12 and 14 days of differentiation (Fig. 1A). Consistently, protein levels for Ndrg1 also increased during 3t3l1 differentiation (Fig. 1B).

Upon mTORC2-dependent activation of Sgk1 kinase, Sgk1 phosphorylates Ndrg1 at five residues (S300, T328, T346, T346 and T366)^13–15^. Importantly, using a phospho-specific antibody, we found that the levels of Ndrg1 phosphorylated by Sgk1 on T346 also increased during adipocyte differentiation (Fig. 1B). These results suggest that Ndrg1 could play an important role in the regulation of adipocyte differentiation, function, or both.

### Ndrg1 is required for adipocyte differentiation

To assess the role of Ndrg1 in adipocyte differentiation, we generated stable 3t3l1 cell lines expressing shRNA against Ndrg1. Expression of shRNA sequence 3 and 4 against Ndrg1 (Ndrg1 sh3 and sh4) resulted in efficient knockdown of Ndrg1 protein (Fig. 1C). Using AdipoRed-mediated triglyceride (TG) quantification and neutral lipid OilRedO staining, we demonstrated that knockdown of Ndrg1 in 3t3l1 cells subjected to 14 days of adipogenic differentiation resulted in roughly 50% reduced lipid accumulation compared to control cells (Fig. 1D and E). Consistently, *de novo* lipid production (lipogenesis) was also markedly reduced in the absence of Ndrg1 (Fig. 1F). Next, we tested whether Ndrg1 deficiency also impacts other functions of adipocytes. To assess the release of TG stored in lipid droplets during the process of lipolysis, we measured the levels of glycerol and free fatty acids (FFAs) released to the cell culture medium from differentiated 3t3l1 cells. Lipolysis was markedly reduced in the absence of Ndrg1 (Fig. 1G and H). Indicating that also adipocytes function is affected by depletion of Ndrg1.

### Ndrg1 promotes the expression of Pparγ and phosphorylation of C/Ebpα

**Figure 2:**
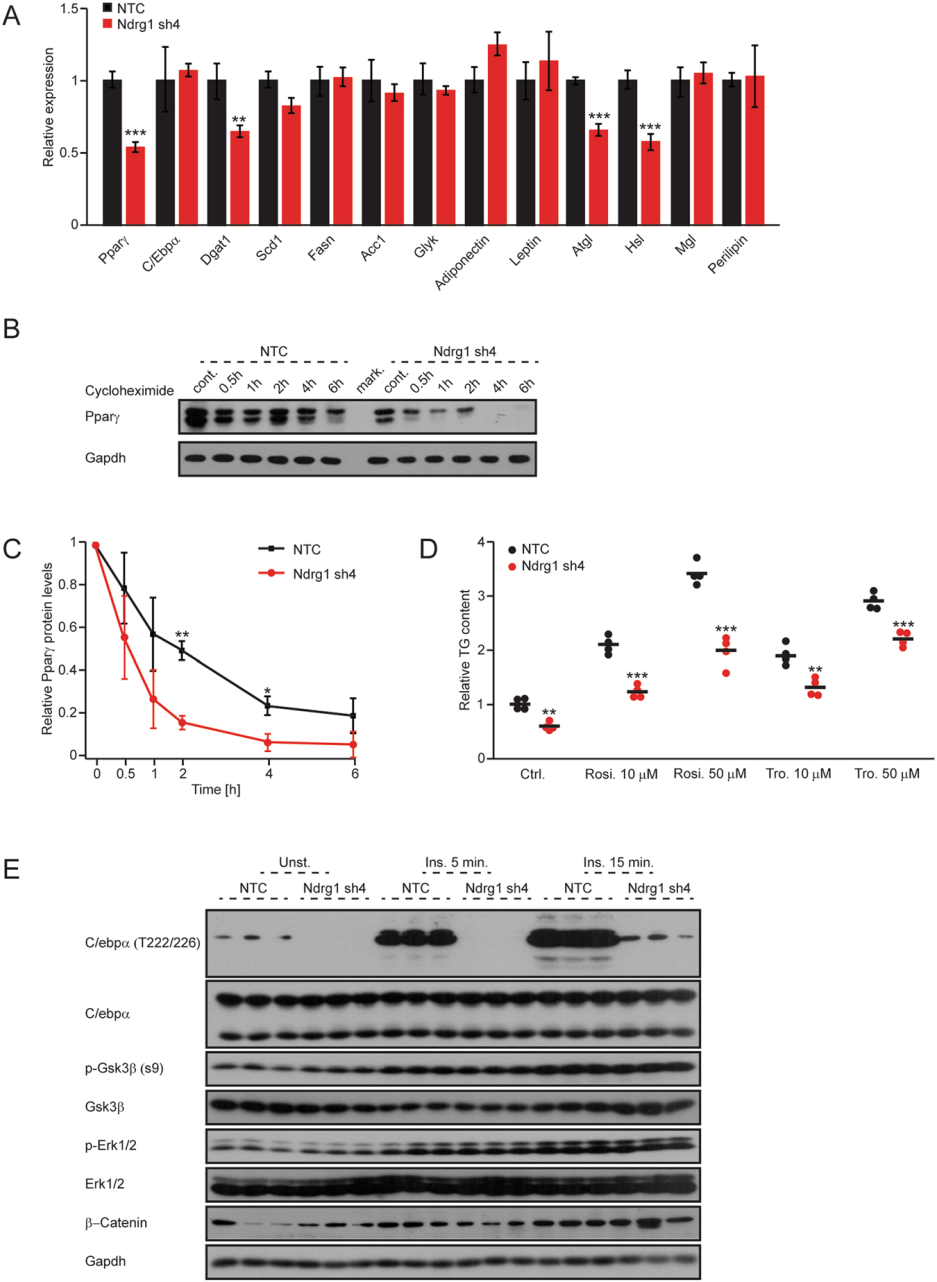
Ndrg1 promotes Pparγ expression and stability as well as C/Ebpα phosphorylation. (**A**) Relative mRNA levels for indicated genes in control and Ndrg1-deficient differentiated 3t3l1 cells. (n = 3, *P < 0.05, **P < 0.01, ***P < 0.001 according to t-test). (**B**) Western blot for Pparγ and Gapdh in control and Ndrg1-depleted differentiated 3t3l1 cells stimulated with cycloheximide for indicated time points. Representative picture was chosen from three biological replicates. (**C**) Quantification of relative amount of Pparγ in cells treated with cycloheximide in relation to the initial (without treatment) levels of Pparγ in respective control and Ndrg1-depleted cells (n = 3, *P < 0.05, **P < 0.01 according to t-test). (**D**) Relative TG content in control and Ndrg1 sh 3t3l1 cells subjected to adipocyte differentiation cocktail with rosiglitazone (Rosi) or troglitazone (Tro). Each data point represents a biological replicate. Stars indicate significance for given parameters between control and Ndrg1-depleted cells. (*P < 0.05, **P < 0.01, ***P < 0.001 according to ANOVA followed by the Post hoc Tukey test). (**E**) Western blot analysis using indicated antibodies in control and Ndrg1-deficient cells stimulated with insulin (Ins.) for the indicated time.

Next, we tested the expression levels of key transcription factors regulating adipocyte differentiation. mRNA levels of Pparγ were markedly reduced in cells expressing shRNA against Ndrg1 (Fig. 2A). Induction of C/Ebpα transcriptional activity is also required for proper adipocyte differentiation^3^, however depletion of Ndrg1 did not influence C/Ebpα mRNA expression. Moreover, the levels of transcriptional targets of Pparγ were reduced in cells depleted from Ndrg1 (Fig. 2A), including diacylglycerol acyltransferase (Dgat) as well as two crucial lipases promoting lipolysis, adipose triglyceride lipase (Atgl) and hormone sensitive lipase (Hsl)^30–32^. Consistently, Pparγ protein levels were markedly reduced in the absence of Ndrg1 (Fig. 2B –without cycloheximide and Supplementary Fig. 1). To test if Ndrg1 also regulates the stability of Pparγ protein, we subjected Ndrg1-deficient 3t3l1 cells to a cycloheximide chase experiment. Cycloheximide treatment resulted in a higher rate of disappearance of Pparγ, in Ndrg1-deficient cells, indicating that Ndrg1 promotes Pparγ stability (Fig. 2B and C). Next, we differentiated Ndrg1-depleted and control 3t3l1 cells in the adipocyte differentiation cocktail supplemented with different concentrations of Pparγ agonists (rosiglitazone, troglitazone). Both agonists markedly induced TG accumulation in 3t3l1 control and Ndrg1-deficient cells. However, treatment with Pparγ agonists did not fully abrogate the difference in TG content between control and Ndrg1-deficient cells (Fig. 2D), suggesting that Ndrg1 could additionally promote the differentiation of adipocytes by targeting other factors than Pparγ.

Previous studies indicated that Ndrg1 might regulate Gsk3β and Erk1/2 activity, as well as the Wnt/β-Catenin pathway^27^. Gsk3β and Erk1/2 have been shown to promote adipogeneis^33, 34^, while expression of β-Catenin suppresses pre-adipocyte differentiation^35^. We tested expression and activation of these proteins in cells depleted of Ndrg1. Western blot analysis revealed that Ndrg1 depletion does not influence the activation or levels of Erk1/2 (Fig. 2E and Supplementary Fig. 1). Similarly, levels of phosphorylated (inactive) and total Gsk3β, as well as β-Catenin were not altered by Ndrg1 silencing (Fig. 2E and Supplementary Fig. 1). Interestingly, Gsk3β promotes phosphorylation of C/Ebpα on threonine 222/226^33^. Therefore, we tested whether Ndrg1 is required for C/Ebpα phosphorylation. We observed that depletion of Ndrg1 suppresses basal and insulin stimulated phosphorylation of C/Ebpα on threonine 222/226, but does not influence total C/Ebpα protein levels (Fig. 2E).

These results suggest that Ndrg1 promotes adipogenesis by enhancing Pparγ expression. In addition, Ndrg1 might also influence C/Ebpα activity through phosphorylation. However, the precise mechanisms of Ndrg1 action needs to be determined.

### Overexpression of Ndrg1 promotes adipocyte differentiation

**Figure 3:**
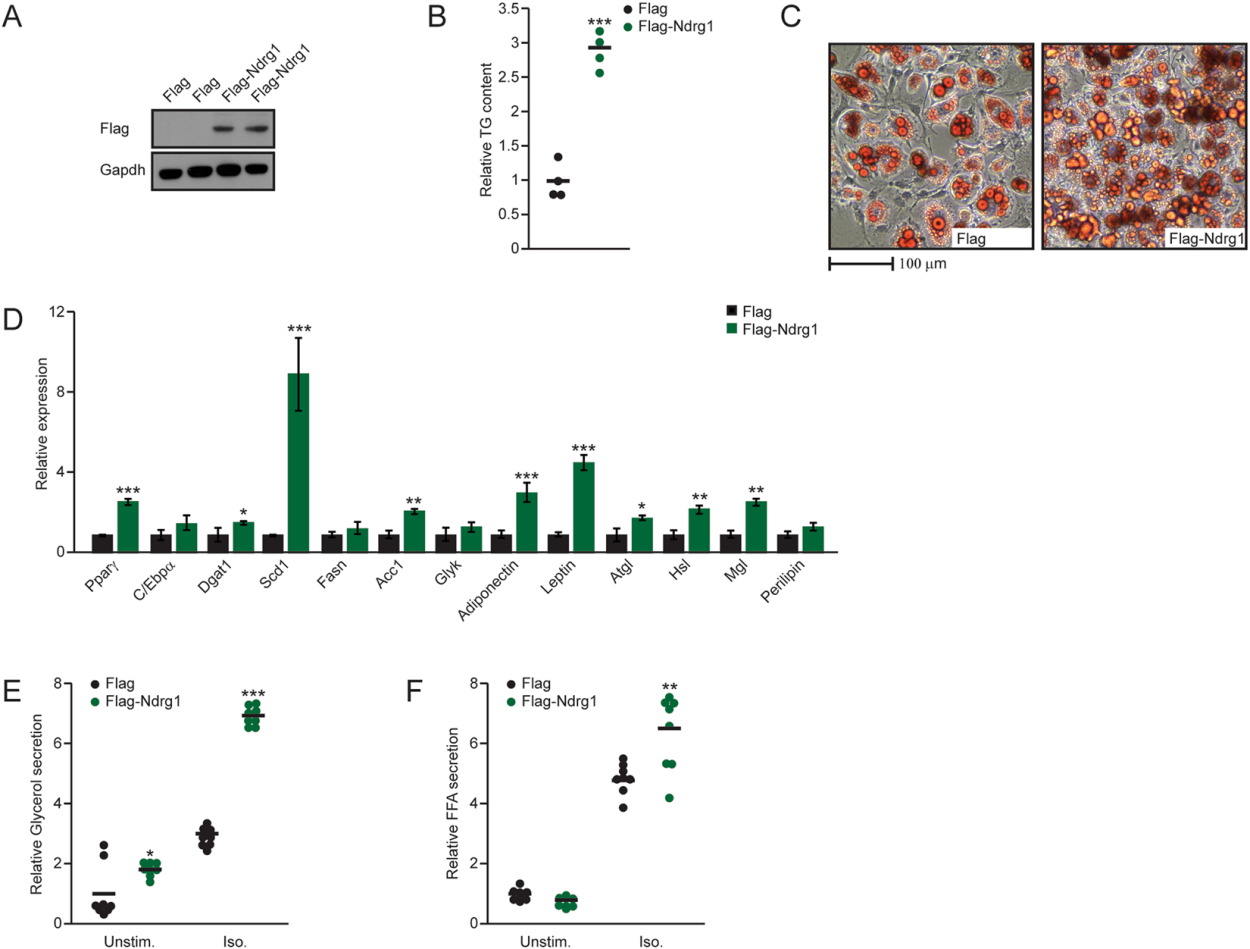
Overexpression of Ndrg1 promotes adipogenesis. (**A**) Western blot using indicated antibodies on extracts isolated from 3t3l1 control and FlagNdrg1-expressing cells. (**B**) Relative TG content and (**C**) neutral lipid staining (OilRedO) of differentiated 3t3l1 control and FlagNdrg1-expressing cells. (**D**) Relative mRNA levels for indicated genes in differentiated control and Ndrg1-overexpressing 3t3l1 cells. (**F**) Relative glycerol and (**G**) FFA levels in medium from differentiated 3t3l1 control cells or Ndrg1-overexpressing cells stimulated with control medium or Iso. Each data point represents a biological replicate (for bar plots n = 3). Stars indicate significance for given parameters between control and Ndrg1- overexpressing cells. (*P < 0.05, **P < 0.01, ***P < 0.001 according to ANOVA followed by the Post hoc Tukey test).

To determine if overexpression of Ndrg1 is sufficient to enhance adipogenesis, we generated 3t3l1 cells ectopically expressing Flag-tagged Ndrg1 (FlagNdrg1). Stable expression of FlagNdrg1 was confirmed by Western blotting (Fig. 3A). Overexpression of Ndrg1 resulted in an over two-fold elevation in the levels of TG in differentiated 3t3l1 cells compared to control cells (Fig. 3B and C). On the molecular level, overexpression of Ndrg1 lead to elevated levels of markers defining adipocyte function. Specifically, we observed increased expression of Pparγ, Dgat, stearoyl-CoA-desaturase (Scd1), acetyl-CoA carboxylase (Acc1), Atgl, Hsl, monoglyceride lipase (Mgl), adiponectin and leptin (Fig. 3D). Moreover, Ndrg1 overexpression enhanced adipocyte function as indicated by increased lipolytic activity (Fig. 3E and F). Altogether, these data suggest that Ndrg1 overexpression promotes adipogenesis and adipocyte function.

### Sgk1-dependent phosphorylation of Ndrg1 on T346 is required for adipogenesis

**Figure 4:**
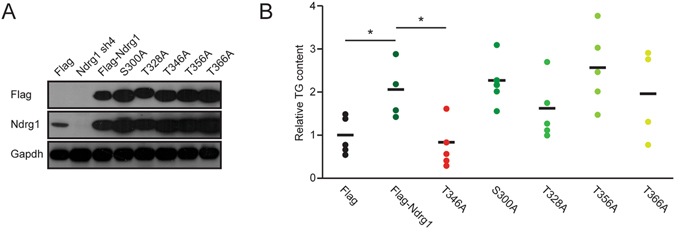
Sgk1-dependent phosphorylation of Ndrg1 is required for its pro-adipogenic function. (**A**) Western blot assessing the levels of expression of different FlagNdrg1 phospho-mutant proteins using the indicated antibodies. (**B**) Relative TG content in differentiated 3t3l1 cells expressing the indicated phospho-mutants of Ndrg1. Each data point represents an average TG content of individually generated and differentiated mixed stable cell populations of 3t3l1 cells expressing different Ndrg1 mutants. (*P < 0.05 according to ANOVA followed by the Post hoc Tukey test).

As a downstream effector of mTORC2, Sgk1 phosphorylates Ndrg1 on multiple sites (S300, T328, T346, T356 and T366)^13–15^. Previous studies indicated that Sgk1 is required for adipocyte differentiation^11^. To test if Sgk1-dependent phosphorylation of Ndrg1 is required for its function, we generated Ndrg1 alanine mutants that cannot be phosphorylated at the respective residues and expressed them in 3t3l1 cells. Western blot analysis confirmed that all mutants were equally overexpressed as the control wild type form of Ndrg1 (Fig. 4A). Next, we subjected all the cell lines to an adipogenic differentiation protocol. As expected, overexpression of the wild type form of Ndrg1 resulted in markedly elevated TG accumulation compared to cells expressing empty vector (Fig. 4B). Overexpression of S300A, T328A, T356A and T366A mutants also resulted in a marked increase in TG accumulation in cells, comparable to wild-type overexpression (Fig. 4B). However, overexpression of the T346A mutant led to markedly lower TG accumulation than cells overexpressing the wild-type form of Ndrg1 (Fig. 4B). These results indicate that Sgk1-dependent phosphorylation of Ndrg1 on T346 promotes adipogenesis.

### Ndrg1 sustains adipocyte function after differentiation

**Figure 5:**
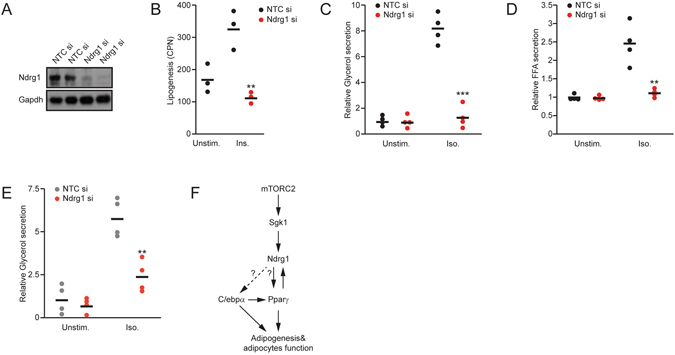
Ndrg1 promotes lipolysis independent of its impact on adipocyte differentiation. (**A**) Western blot for the indicated proteins in differentiated 3t3l1 cells transfected with control or Ndrg1 siRNA. (**B**) Lipogenesis rate in control siRNA and Ndrg1 siRNA transfected cells (Ndrg1 si), unstimulated (unstim.) or upon insulin (Ins.) stimulation. (**C**) Relative glycerol and (**D**) FFA levels in medium from differentiated 3t3l1 control cells or Ndrg1 siRNA-treated cells stimulated with control medium or Iso. (**E**) Relative glycerol levels in adipocytes differentiated from primary stroma-vascular cells isolated from subcutaneous fat pads and transfected with non-targeted control (NTC) or Ndrg1 siRNA. Each data point represents a biological replicate. Stars indicate significance for given parameters between control and Ndrg1-depleted cells. (*P < 0.05, **P < 0.01, ***P < 0.001 according to ANOVA followed by the Post hoc Tukey test). (**F**) Model of Ndrg1 action in adipocytes. mTORC2 activates Sgk1 kinase, which phosphorylates Ndrg1 on T346. Phosphorylated Ndrg1 increases expression of Pparγ through an unknown mechanism to promote adipogenesis and adipocyte function. In a positive feedback loop, Pparγ also promotes Ndrg1 expression and stability. Additionally, Ndrg1 promotes C/Ebpα phosphorylation, which might influence its activity.

To test if Ndrg1 plays a role in the regulation of mature adipocyte function, we knocked down Ndrg1 using transient siRNA transfection in fully differentiated 3t3l1 cells. Effective knockdown of Ndrg1 was confirmed by Western blot (Fig. 5A). Consistent with our previous results, siRNA-mediated silencing of Ndrg1 resulted in a reduced rate of lipogenesis (Fig. 5B). Moreover, knockdown of Ndrg1 decreased the rate of isoproterenol-induced lipolysis in 3t3l1 cells, as indicated by a more than two-fold reduction in the concentration of FFAs and a more than three-times reduced levels of glycerol in the medium compared to control cells (Fig. 5C and D). To confirm these results, we knocked down Ndrg1 in adipocytes differentiated from primary stromal vascular cells isolated from wild type mice. Ndrg1 knockdown in primary cells resulted in a similarly reduced rate of lipolysis (Fig. 5E), demonstrating that the observed phenotype is not restricted to 3t3l1 cells.

## Discussion

Our results indicate that Ndrg1 is both activated during and required for adipogenesis. Moreover, in fully differentiated adipocytes Ndrg1 stimulates lipolysis. Ndrg1 promotes adipocyte formation partially by enhancing expression of the crucial pro-adipogenic transcription factor Pparγ. However, Ndrg1 might also regulate C/Ebpα adipogenic activity (Fig. 5F).

Ndrg1 is a distal effector of mTORC2 activation in cells^9^. Disruption of mTORC signaling by silencing its component rictor results in reduced adipocyte differentiation^6^. Sgk1 kinase is directly activated by mTORC2; consistently, its depletion results in attenuated adipogenesis^9–12^. In agreement with these data, we showed that Sgk1-dependent phosphorylation of Ndrg1 is required for its pro-adipogenic function. However, previous results indicate that the mTORC2 signaling module suppresses lipolysis^36^. As our results indicate that Ndrg1 promotes lipolysis, mTORC2 must therefore suppress lipolysis by utilizing an Ndrg1-independent mechanism. Of note, expression of Atgl and Hsl, which are targets of Pparγ^31, 32^ were reduced in the absence of Ndrg1. These results explain the attenuated lipolysis rate in the absence of Ndrg1. Moreover, our results might indicate that defective lipolysis in cells deficient for Ndrg1 may be a direct consequence of reduced differentiation of these cells. We showed that Pparγ activation increases Ndrg1 expression. On the other hand, our results indicate that Ndrg1 promotes expression and stability of Pparγ. Therefore we postulate that Ndrg1 acts as a component of a positive feedback loop promoting Pparγ action.

However, agonist-mediated activation of Pparγ did not completely rescue the adipogenesis defect in Ndrg1-deficient cells. This might be caused by the lower protein levels of Pparγ in cells depleted of Ndrg1. Alternatively, this might indicate that Ndrg1 also regulates other components of the molecular machinery defining adipocyte differentiation. In fact, we observed that phosphorylation of C/Ebpα on threonines 222/226 is markedly reduced in the absence of Ndrg1. Threonines 222 and 226 on C/Ebpα were originally identified as targets for Gsk3β-dependent phosphorylation^33^. However, a recent study indicated that C/Ebpα is a poor substrate for Gsk3β^37^. In fact, our results suggest that Ndrg1 does not influence the total levels of Gsk3β or its phosphorylated (inactive) form. This suggests that Ndrg1 modulates C/Ebpα phosphorylation by influencing Gsk3β activity in another manner or by utilizing a Gsk3β-independent mechanism. Also, the impact of C/Ebpα T222/T226 phosphorylation on adipogenesis has not been directly assessed. Phosphorylation on these sites was originally proposed to enhance adipogenesis^33^, but a recent study indicates that it might reduce C/Ebpα activity^37^.

Previous studies implicated Ndrg1 in the regulation of Wnt/β-Catenin as well as Erk1/2 action^27^. Our results indicate that Ndrg1 does not influence any of these pathways in adipocytes. Therefore, we postulate that Ndrg1 primarily promotes adipogenesis by enhancing expression of Pparγ. However, Ndrg1 might also influence other adipogenic factors and the exact mechanism of its action needs to be defined further.

## Methods

### Pre-adipocyte culture and differentiation

3t3l1 preadipocytes were cultured in DMEM supplemented with 10% fetal calf serum (FCS), and 40 μg/ml Gentamicin at 37 °C in a humidified atmosphere. For differentiation, 3t3l1 were grown to confluency. Two days post-confluency, the cells were switched to medium containing 10% fetal bovine serum, 1.5 µg/ml insulin, 0.5 mM 3-isobutyl-1-methylxanthine (IBMX) and 1 μM Dexamethasone (Dex) for 48 hours. This step was repeated for an additional 48 hours and then the induced cells were incubated with “Differentiation Medium” (DMEM supplemented with 10% FBS and 1.5 µg/ml insulin) for up to 10 days to achieve differentiation to adipocytes. In the indicated experiments, Pparγ agonists (rosiglitazone or troglitazone) were supplemented to the differentiation medium at indicated concentrations throughout the differentiation process. Fresh agonists were added to the medium every 48 h. All compounds were purchased from Sigma-Aldrich if not specified.

Stromal vascular cells (SVCs) containing pre-adipocytes were isolated from adipose tissue Briefly, subcutaneous white adipose tissue were collected from wild type BL6 mice and cut into small fragments with a scalpel. The sliced SVC fractions were treated with 2 mg/ml Collagenase D (Roche) in PBS containing 5 mM CaCl_2_, 1% BSA and P/S with shaking (300 rpm) at 37 °C for 40 minutes. After digestion of adipose tissue, the solution was filtered through a cell strainer with 45 μm pore size and centrifuged at 1250 rpm for 10 minutes to collect SVCs. SVCs were cultured in complete medium (DMEM/F12 containing 10% FBS, 1% sodium pyruvate (SP), 1% non-essential amino acids (NEAA) and P/S) until two days post-confluent and transferred into induction medium (DMEM/F12 containing 10% FBS, 1% SP, 1% NEAA and P/S, 0.2 μM Indomethacin, 0.5 μM Rosiglitazone, 0.5 mM IBMX and 1 μM Dex) for two times 48 hours. Then, the SVC cells were cultured into complete medium with 1.5 μg/mL insulin for up to 6 additional days.

### siRNA transfection

Fully differentiated 3t3l1 cells were transfected using Dharmafect Duo transfection reagent with siRNA against Ndrg1 or NonTarget control (Dharmacon) according to the manufacturer’s protocol. Cells were used for experiments 48 hours post transfection.

### Generation of stable knockdown and overexpression of wild type and mutant Ndrg1

shRNA against Ndrg1 was introduced into 3t3l1 cells using lentiviral particles. Specific shRNA sequences (available in Supplementary Table 1) were cloned in pGIPZ vector was described previously^38^. The infected 3t3l1 cells were then selected by puromycin treatment (5 μg/ml). The pBabe-Puro vector was used for retrovirus-driven Flag-Ndrg1 expression. A full length coding sequence of Ndrg1 was introduced between EcoRI and BamHI restriction sites of this vector. Platinum-E (*Plat*-*E*) cell were utilized to produce retroviral particles. Infected 3t3l1 cells were selected with puromycin (5 μg/ml). Ndrg1 mutants were generated using the site-directed mutagenesis kit from New England Biolabs according to the manufacturer’s protocol.

### Analyses of mRNA expression and protein levels

Quantitative polymerase chain reaction (qPCR) was performed using SYBR green Universal PCR master mix (Roche). mRNA was isolated using trizol reagent (Invitrogen). cDNA was generated using First Strand cDNA synthesis kit (Thermo Scientific). Primers used in this study are available in Supplementary Table 1. Western blot analyses were performed according to standard procedure. All antibodies were obtained from Cell Signaling Technology. For indicated experiments, cycloheximide (Sigma, 50 μg/ml concentration) was added to the culture medium to block protein translation. The levels of indicated proteins were assessed by western blot after the indicated time points.

### Quantification of lipid levels and DNA content

Double staining of AdipoRed reagent (Lonza, Basel, Switzerland) and Hoechst 33342 (Invitrogen) was utilized to quantify the intracellular lipid accumulation in relation to DNA content. Oil-Red O staining (Sigma) was used to visualize TG in cells. To assay lipolysis, cells were starved for 2 hours in phenol red-free DMEM supplemented with 0.5% BSA, followed by stimulation with 10 μM isoproterenol (iso) (Sigma) for 2 hours in the same medium. FFAs in the medium were quantified using NEFA-reagent from Wako Chemicals and glycerol was quantified using the specific reagent from Sigma.

### Lipogenesis assay

For determination of *de novo* lipogenesis, 3T3L1 cells were incubated with ^3^H-glucose (1 µCi/mL, Perkin Elmer) in the presence or absence of insulin for 3 h. Cells were washed twice with PBS and lysed in 0.1 N HCl. Lipids were then extracted with chloroform/methanol (2:1, v/v). The lipid-containing chloroform phase was used for liquid scintillation counting.

### Statistical analysis

For multiple comparisons, one way analysis of variance (ANOVA) followed by the Post hoc Tukey test was used. For determination of significance between two experimental groups T-Test was used. Significance was accepted at the level of 0.05. Exact statistics are indicated for each figure.

## Electronic supplementary material

Supplementary information
