# Supplementary material for: Ndrg1 promotes adipocyte differentiation and sustains their function
Source: Sci Rep. 2017 Aug 3;7:7191. doi: 10.1038/s41598-017-07497-x (PMC5543145; doi:10.1038/s41598-017-07497-x)
Supplement: Supplementary file 1 — Supplementary information [file 41598_2017_7497_MOESM1_ESM.pdf]

Supplementary information:

Ndrg1 promotes adipocytes differentiation and sustains their function

Kai Cai, Rabih El-Merahbi, Mona Loeffler, Alexander E. Mayer and Grzegorz Sumara

Rudolf Virchow Center for Experimental Biomedicine, University of Würzburg, Josef-Schneider-Str. 2, Haus D15, D-97080 Würzburg, Germany

Supplementary Table 1

QPCR primers:

**Gene Name**	**Gene Symbol**	**Forward sequence**	**Reverse Sequence**
Peroxisome proliferator-activated receptor-γ	Pparγ	GGAAGACCACTC GCATTCCTT	GTAATCAGCAACC ATTGGGTCA
Fatty acid synthase	Fasn	GGAGGTGGTGAT AGCCGGTAT	TGGGTAATCCATA GAGCCCAG
Hormone-sensitive lipase	Hsl	TTCTCCAAAGCAC CTAGCCAA	TGTGGAAAACTAA GGGCTTGTTG
Adipose triglyceride lipase	Atgl	CAACGCCACTCA CATCTACGG	GGACACCTCAATA ATGTTGGCAC
Glycerol kinase	Glyk	CGGAGACCAGCC GTGTTAAG	GTCCACTGCTCCCA CCAATG
acetyl-Coenzyme A carboxylase alpha	Acc1	GA CAG ACT GAT CGC AGA GAA AG	TGG AGA GCC CCA CAC ACA
stearoyl-Coenzyme A desaturase 1	Scd1	CCC AGT CGT ACA CGT CAT TTT	CAT CAT TCT CAT GGT CCT GCT
diacylglycerol O-acyltransferase	Dgat1	GTG CCA TCG TCT GCA AGA TT	CTG GAT AGG ATC CAC CAG GA
Adiponectin	Adiponectin	CCAACCTGCACA AGTTCCCTT	TGTTCCTCTTAATC CTGCCCA
N-Myc downstream regulated gene 1	Ndrg1	ATGTCCCGAGAG CTACATGAC	CCTGCTCCTGAACA TCGAACT
Monogliceride lipase	Mgl	ACCATGCTGTGAT GCTCTCTG	CAAACGCCTCGGG GATAACC
CCAAT/enhancer binding protein (C/EBP) alpha	C/Ebpα	AAACAACGCAAC GTGGAGA	GCGGTCATTGTCAC TGGTC
Leptin	Leptin	GTGGCTTTGGTCC TATCTGTC	CGTGTGTGAAATGT CATTGATCC
Perilipin 1	Perilipin 1	GGG ACC TGT GAG TGC TTC C	GTA TTG AAG AGC CGG GAT CTT TT

shRNA sequences:

Ndrg1 seq1	TGCTGTTGACAGTGAGCGTGCTGTTGACAGTGAGCGACAGGATCTTG GAGTTGCTAGATAGTGAAGCCACAGATGTATCTAGCAACTCCAAGAT CCTGGTGCCTACTGCCTCGGACTTCAAGGGGCTAGAATTC
Ndrg1 seq2	TGCTGTTGACAGTGAGCGTGCTGTTGACAGTGAGCGCTCGCTGGTTCT TCAGAGAGAATAGTGAAGCCACAGATGTATTCTCTCTGAAGAACCAG CGATTGCCTACTGCCTCGGACTTCAAGGGGCTAGAATTC
Ndrg1 seq3	TGCTGTTGACAGTGAGCGTGCTGTTGACAGTGAGCGCCGGCAAGGAG GAGATACACAATAGTGAAGCCACAGATGTATTGTGTATCTCCTCCTTG CCGATGCCTACTGCCTCGGACTTCAAGGGGCTAGAATTC
Ndrg1	TGCTGTTGACAGTGAGCGTGCTGTTGACAGTGAGCGCTCCGTGCTTGC

seq4	TTGCTCATTATAGTGAAGCCACAGATGTATAATGAGCAAGCAAGCAC GGAATGCCTACTGCCTCGGACTTCAAGGGGCTAGAATTC
Ndrg1 seq5	TGCTGTTGACAGTGAGCGTGCTGTTGACAGTGAGCGCAGGGATCAGTT TACCTGCCAATAGTGAAGCCACAGATGTATTGGCAGGTAAACTGATCC CTTTGCCTACTGCCTCGGACTTCAAGGGGCTAGAATTC

Supplementary Fig. 1 Ndrg1 promotes Pparγ abundance.
Western blot for indicated proteins in control and Ndrg1-depleted differentiated 3t3l1 cells stimulated with indicated substances.

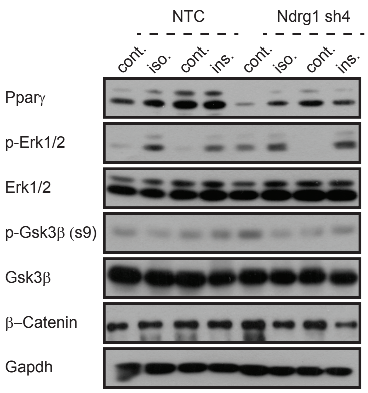
NTC
Ndrg1 sh4
cont.
iso.
cont.
ins.
cont.
iso.
cont.
ins.
Pparγ
p-Erk1/2
Erk1/2
p-Gsk3β (s9)
Gsk3β
β–Catenin
Gapdh
